# The UCSC Genome Browser database: 2017 update

**DOI:** 10.1093/nar/gkw1134

**Published:** 2016-11-28

**Authors:** Cath Tyner, Galt P. Barber, Jonathan Casper, Hiram Clawson, Mark Diekhans, Christopher Eisenhart, Clayton M. Fischer, David Gibson, Jairo Navarro Gonzalez, Luvina Guruvadoo, Maximilian Haeussler, Steve Heitner, Angie S. Hinrichs, Donna Karolchik, Brian T. Lee, Christopher M. Lee, Parisa Nejad, Brian J. Raney, Kate R. Rosenbloom, Matthew L. Speir, Chris Villarreal, John Vivian, Ann S. Zweig, David Haussler, Robert M. Kuhn, W. James Kent

**Affiliations:** 1 Genomics Institute, University of California Santa Cruz, Santa Cruz, CA 95064, USA; 2 Emory University School of Medicine, Atlanta, GA 30322, USA; 3 Howard Hughes Medical Institute, University of California Santa Cruz, CA 95064, USA

## Abstract

Since its 2001 debut, the University of California, Santa Cruz (UCSC) Genome Browser (http://genome.ucsc.edu/) team has provided continuous support to the international genomics and biomedical communities through a web-based, open source platform designed for the fast, scalable display of sequence alignments and annotations landscaped against a vast collection of quality reference genome assemblies. The browser's publicly accessible databases are the backbone of a rich, integrated bioinformatics tool suite that includes a graphical interface for data queries and downloads, alignment programs, command-line utilities and more. This year's highlights include newly designed home and gateway pages; a new ‘multi-region’ track display configuration for exon-only, gene-only and custom regions visualization; new genome browsers for three species (brown kiwi, crab-eating macaque and Malayan flying lemur); eight updated genome assemblies; extended support for new data types such as CRAM, RNA-seq expression data and long-range chromatin interaction pairs; and the unveiling of a new supported mirror site in Japan.

## INTRODUCTION

With the revolutionary rate at which vast amounts of biological data are now being generated, new technologies and resources that can accommodate computational challenges are constantly in demand. The UCSC Genome Browser (1) continually strives to meet the needs of biological researchers in the face of exponential growth. This update gives a brief overview of the Genome Browser tools and data, and describes what has been added, changed, and improved since the previous 2016 update (2).

### Genome assemblies

From its original focus on the early drafts of the human genome, the Genome Browser database now offers genomic data for nearly 100 organisms, many with multiple assemblies. Available as genome browsers and as downloadable data, this year there are three new species assemblies available (brown kiwi, crab-eating macaque, Malayan flying lemur) and new assembly versions for the following eight species: *C. elegans*, cat, chicken, gray mouse lemur, platypus, rhesus macaque monkey, western clawed frog, and western gorilla (3–8). For a detailed list of new and updated UCSC-hosted genomes, see Table 1.

**Table 1.** New and updated UCSC-hosted genomes, 2015–2016

Scientific name (common name)	Assembly date	UCSC ID	Sequencing center, NCBI accession ID, Assembly name
New Species			
Apteryx australis mantelli (brown kiwi)	June 2015	aptMan1	Max-Plank Institute for Evolutionary Anthropology GCF_001039765.1 AptMant0
Macaca fascicularis (crab-eating macaque)	June 2013	macFas5	Washington University (WashU) GCA_000364345.1 Macaca_fascicularis_5.0
Galeopterus variegatus (malayan flying lemur)	June 2014	galVar1	Washington University (WashU) GCF_000696425.1 G_variegatus-3.0.2
Updated Species			
Felis catus (domestic cat)	November 2014	felCat8	International Cat Genome Sequencing Consortium GCA_000181335.3 Felis_catus_8.0
Caenorhabditis elegans (C. elegans)	February 2013	ce11	C. elegans Sequencing Consortium WBcel235 GCA_000002985.3 WBcel235
Xenopus tropicalis (tropical clawed frog)	September 2012	xenTro7	US DOE Joint Genome Institute (JGI-PGF) GCA_000004195.2 Xtropicalis_v7
Gorilla gorilla (western gorilla)	December 2014	gorGor4	Wellcome Trust Sanger Institute GCA_000151905.3 gorGor4
Microcebus murinus (gray mouse lemur)	May 2015	micMur2	Broad Institute and Baylor College of Medicine GCA_000165445.2 Mmur_2.0
Ornithorhynchus anatinus (platypus)	February 2007	ornAna2	Washington University Genome Sequencing Center GCA_000002275.2 Ornithorhynchus_anatinus-5.0.1
Macaca mulatta (rhesus monkey)	November 2015	rheMac8	Baylor College of Medicine Genome Sequencing Center GCF_000772875.2 Mmul_8.0.1
Gallus gallus (chicken)	November 2011	galGal5	Chicken Genome Consortium (ICGC) GCA_000002315.2 Gallus_gallus-5.0

Genome sequences and annotations for all UCSC-hosted assemblies can be downloaded from http://hgdownload.soe.ucsc.edu/. Downloadable annotations for UCSC-hosted assemblies generally include GenBank sequence alignments (9), repeat annotations using RepeatMasker (Smit *et al., RepeatMasker Open-4.0* at http://repeatmasker.org) and Tandem Repeats Finder (10), soft and hard masked assembly sequences, chromosome sizes, and more.

### Genome browser annotation data tracks

Assemblies for all species contain a set of basic annotations (‘tracks’), some of which are automatically generated by UCSC (e.g., assembly and gaps, the percentage of guanine and cytosine bases, repeat regions). Popular research species such as human and mouse are much more richly annotated, with tracks showcasing data from international sources.

The UCSC Genome Browser team regularly updates data tracks and adds new annotations. Although some annotations are mapped from the previous assembly (particularly in recent human assemblies), most data sets are obtained from the original data providers or generated from original sources. All assemblies for which GenBank provides cDNA sequences receive weekly automatic updates for relevant tracks (e.g., RefSeq Genes, ESTs, mRNAs). Other new and updated data for most assemblies include genome alignment data (chain and net tracks), predicted Augustus Genes (11,12) for all new assembly databases and Ensembl Genes (13).

The following descriptions are a sampling of new and updated data tracks in the Genome Browser database, grouped by track category. For a complete list of new and updated tracks released in this last year, see Supplementary Table S1.

#### Mapping and sequencing

Tracks in this group annotate the foundational structure of the assembly and constructed contigs. Beyond automatically updated tracks, most of the prominent new tracks in the Mapping and Sequencing category this year were added to the human GRCh38/hg38 assembly. These include a new fluorescent *in situ* hybridization (FISH) clones track (14) lifted from NCBI36/hg18, and a new track that annotates STS markers (15,16). A Clone Ends track was added showing mapped clone end libraries for mouse (GRCm38/mm10) and rat (RGSC_6.0/rn6) from NCBI's Clone DB (17).

#### Genes and gene predictions

This group includes computationally predicted gene sets (e.g., Ensembl (13), Augustus), high-quality manually curated and evidence-based automated gene predictions for the human and mouse genomes from the GENCODE project (18), and subsets from NCBI such as manually curated sets from the Reference Sequence collection (RefSeq) (19). This year there were several major gene set additions and updates, primarily to the latest human and mouse assemblies. Existing Ensembl gene annotations were updated to version 81, and new Ensembl tracks were added to several assemblies: *Drosophila* (dm6), rat (rn6) and zebrafish (danRer10). Two major gene sets (GENCODE Genes, UCSC Genes) were updated, as described below.

##### GENCODE genes

The GENCODE Genes set is now the default gene set for the latest GRCh38/hg38 human assembly, replacing the previous default set, UCSC Genes. This transition was made to reduce the number of competing gene transcript sets used by the bioinformatics community, which have caused confusion in downstream analyses due to subtle differences among the sets. As of September 2016, the browser displays the v24 version, released by GENCODE in August 2015. The v24 gene set has increased by 2604 new transcripts to a total of 197 782 total transcripts. Only the Basic subset is displayed in the Genome Browser by default; the underlying database tables contain the Comprehensive set, which can be viewed by clicking the ‘show comprehensive set’ checkbox on the track controls page. The database schema retains most of the same table names (starting with ‘known’ or ‘kg’). Every transcript has a UCSC identifier (e.g., uc001abz.5) in addition to the GENCODE identifier (e.g., ENST00000327044.6).

##### UCSC genes

The UCSC Genes set (20) has been updated for the mm10 mouse assembly. The new release increased by 515 new transcripts to a total of 63 759 total transcripts, 95% of which remained the same between versions. The total number of canonical genes has increased by 121 to a total of 33 079.

#### Phenotype and literature

This group contains tracks linking to phenotype references and literature (e.g., published articles) in which referenced sequences or identifiers are mapped to the assembly. The GRCh37/hg19 human assembly is currently the most comprehensively annotated with 21 major tracks, displaying data related to genetic phenotypes such as Online Mendelian Inheritance in Man (OMIM) allelic variants and positions of OMIM genes (21) as well as variants and copy number variants from the National Institutes of Health (NIH) funded ClinVar (22) and ClinGen (23) datasets. One highlight in this category is the addition of a super-track showing the genomic mapping of biomedical sequences submitted as part of patent application documents worldwide, obtained from the Cambia Lens PatSeq database (24) and aligned to the human (hg19), mouse (mm10) and Ebola virus (eboVir3) genome assemblies (25).

Other new track additions for the recent human assemblies include new copy number variant tracks for CNV Developmental Delay and ClinGen Benign Aggregate, both for hg38 and hg19. In hg38's new non-coding RNA track (lifted from hg19), four different types of RNA data are positioned in three different sub-tracks: sno/miRNAs, tRNAs (also added as a new track in hg19), and lincRNAs (26–29).

As of 2016, proprietary OMIM data can now be accessed on a per-chromosome basis from the Table Browser and the Data Integrator; whole genome downloads are not available, per agreement with OMIM.

#### Expression

**Figure 1.**
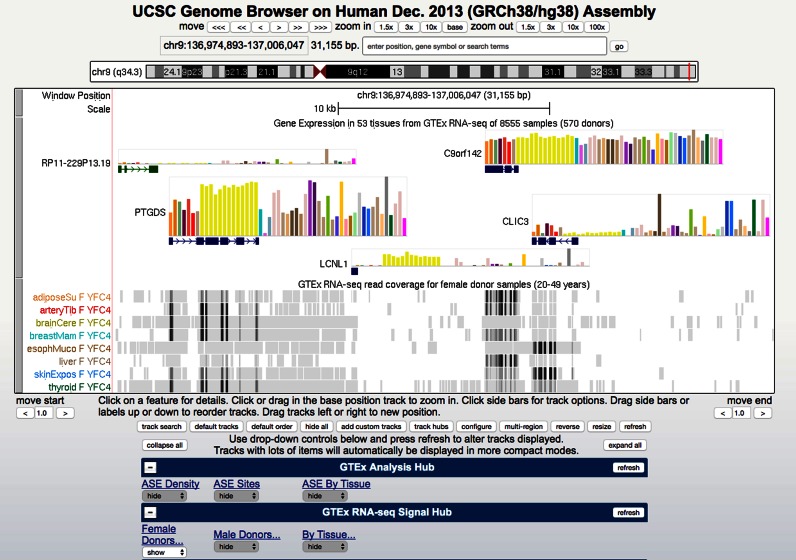
GTEx tracks in the UCSC Genome Browser. This image shows a 31 kb region of chromosome 9 where GENCODE annotates four protein-coding genes showing different patterns of tissue-specific gene expression in GTEx tissue samples. The GTEx Gene Expression track displays a bar graph for each gene, where bar color indicates the tissue assayed and bar height depicts the median expression level across all samples in reads per kilobase of transcript per million mapped reads (RPKM). Tissues are identified on mouseover and the full legend is shown on the track description and details pages. The GTEx transcript model used to quantify expression level is displayed below the graph, colored by gene class using GENCODE conventions. The GTEx RNA-seq tracks show per sample read density; in this image, eight tissues from a single GTEx donor. The GTEx Analysis Hub is also connected and therefore is visible in the track menu, but no tracks are selected for display. Tissue colors in all GTEx tracks are assigned using GTEx project conventions.

This category contains tracks related to the synthesis of functional gene products. A new gene expression track based on data from the NIH Genotype-Tissue Expression (GTEx) project (30,31) was released for the most recent human assemblies, hg38 and hg19. The GTEx track displays tissue-specific gene expression based on RNA-seq in 53 tissues from 570 donors obtained from the GTEx midpoint data release (V6, October 2015). This browser track uses a new bar graph display with a graph shown for each gene, and a colored bar indicating each tissue assayed (Figure 1). The ‘squish’ view colors the gene by most highly expressed tissue. A box plot showing the range of expression levels across samples is displayed on the details page for each gene in the GTEx track, and also on the details page for that gene on the browser's default gene set.

Additional GTEx expression data are available in two UCSC-hosted track hubs. The first is the GTEx RNA-Seq Signal hub, hosting ∼7000 tracks, one for each tissue sample in the midpoint data release. The second is the GTEx Analysis hub, which currently hosts summary tracks of allele-specific expression (per-tissue and cross-tissue). GTEx gene expression has also been incorporated in the Gene Sorter tool, replacing GNF Atlas2 as the default gene expression set.

#### Regulation

This group contains tracks related to regulation of gene expression, including a wide range of mechanisms used to increase or decrease the production of gene products. The most notable tracks in this category are found primarily on the hg19 assembly, representing data from the ENCyclopedia Of DNA Elements (ENCODE) project (32). The UCSC Genome Browser offers ENCODE data through 2012 (33). The entire collection of ENCODE results from 2007 until present day are available from the ENCODE Project Portal (http://encodeproject.org) (34) hosted by Stanford University. The major integrated ENCODE tracks have been mapped to hg38; however, the bulk of annotations remain on hg19.

#### Comparative genomics

Conservation tracks combine multi-species genome-wide alignments and estimated evolutionary conservation and acceleration scores to enable visualization and analysis of evolutionary processes. In September 2015, a new 100-vertebrate conservation track was released on the hg38 human assembly that compares pairwise alignments for 100 species from a variety of clades. Another addition is the *C. elegans* (ce11) 26-species conservation track, which shows the multiple alignments and measurements of evolutionary conservation for 26 nematode species.

#### Variation

Major variant annotation tracks include small variations from NCBI's dbSNP database (35), including tracks for older dbSNP releases in human assemblies, along with variant calls and high-confidence regions from the 1000 Genomes Consortium (36).

##### New dbSNP Build 146 and Build 147

Data from NCBI's dbSNP Builds 146 and 147 have been added to the two most recent human assemblies (hg38, hg19), and Build 146 annotations were also added to the mm10 mouse assembly. Both builds 146 and 147 contain >150 million variants for human.

##### New phase 3 data of the 1000 Genomes Project

Data from Phase 3 of the 1000 Genomes Project (representing 2504 individuals from 26 populations worldwide) are now available for the hg19 human assembly. These data include nearly 90 million single nucleotide variants (SNVs), insertions/deletions (indels), and structural variants (SVs). For each variant, a detail page incorporates links to the variant in the dbSNP database, quality scores and allele frequency information for various populations. In addition to variant calls, the ‘Paired-end Accessible Regions’ tracks encompass genomic regions estimated to be accessible to next generation sequencing technologies using short paired-end reads, based on depth and quality of mapped reads.

### Home and gateway pages

While the home page of the UCSC Genome Browser is the front door to the online browser, the gateway page is the entrance to visualizing all genomes hosted by the browser, providing opportunities to access 96 unique species (all eukaryotic, except for one virus), whose assembly versions combine to a total of 175.

Both the home page and the gateway page have been redesigned this year to be more interactive and intuitive. The new home page (Supplementary Figure S1) includes more graphics, less text, and better access to Genome Browser tools and content, such as the ability to access popular organisms from the site-wide menu. The newly designed gateway page (Supplementary Figure S2) features an autocomplete search for both UCSC-hosted and public assembly hubs, an interactive phylogenetic tree menu for selecting species, quick access shortcuts to popular browsers, and a new style and color scheme. From both the gateway page and the genome browsers, the ability to search genomes by submitting a chromosomal position, gene symbol, or search term remains a much-used feature. For the human genome, the hg38 assembly is now the default; the previous default assembly, hg19, can be quickly selected from the Genomes menu in the site-wide top menu bar.

### Visualizing genomes: genome browser tracks display

The Genomes Browser tracks display can be seen as the ‘heart’ of the browser. The selected reference genome assembly provides navigational structure for browsing sequence alignments and annotations. The fast, responsive interface shows details from the nucleotide base level out to whole-chromosome perspectives. Major features of the browser display include a powerful search for all data tracks; track configuration and reordering; reverse strand view; and a variety of navigation options to zoom, pan, or quickly jump between features.

#### New multi-region display feature

**Figure 2.**
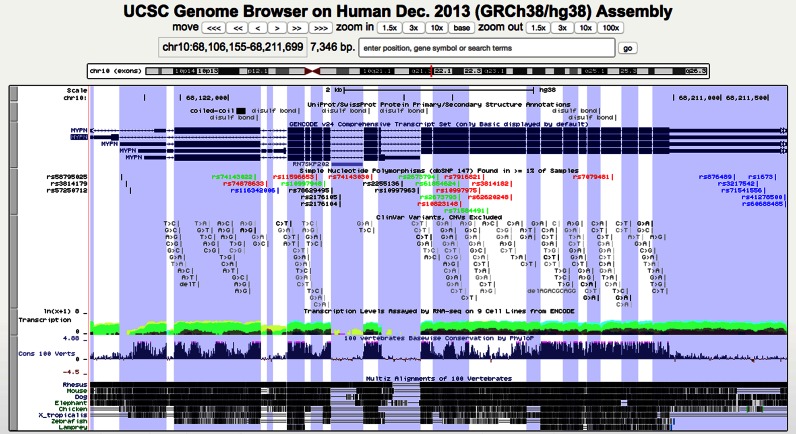
Multi-Region display in the UCSC Genome Browser. This figure displays the MYPN gene in the UCSC Genome Browser using the new multi-region display mode with the ‘exon-only’ option and ‘highlight alternating regions’ enabled. Exonic regions are calculated using the GENCODE v24 Comprehensive Transcript Set on the GRCh38/hg38 human genome assembly and are highlighted in alternating white and blue backgrounds. Intergenic and intronic regions that do not overlap with exons in other transcripts are removed from the display.

The multi-region display configuration was developed to omit introns or intergenic regions from the display, resulting in a Genome Browser display with exon-only or gene-only views (Figure 2), much like a handheld paper fan where portions of the design are hidden behind creased folds. This custom viewing mode can be launched from the browser multi-region button, from the ‘View’ menu on the navigation bar, or (in the case of the exon-only view) by using the keyboard shortcut ‘e v’ while on the graphic browser window.

From the multi-region configuration window, clicking the checkbox to turn on alternating region highlights will help differentiate alternating regions; every other region will appear with a tinted hue, thus creating a series of vertical stripes in the graphic browser window as the genome is ‘sliced’ to display only the defined regions.

The multi-region configuration window offers several display choices: exon-only, gene-only, alternate haplotype view, or custom BED regions. For the exon-only and gene-only modes, the browser automatically chooses which gene track to use as the source for the region coordinates, and the user may configure the padding between features. Available on human assemblies hg17 and newer is the option to display an alternate haplotype within the context of the reference genome (Supplementary Figure S3). A particularly powerful feature of the multi-region configuration is the option to specify the URL of a remotely hosted BED (Browser Extensible Data) file containing coordinates that define custom genomic regions, even those that span chromosomes.

#### New density graph feature

Another new visualization option for tracks is a density graph track display, useful for viewing tracks with many items in a large region (e.g., short-read sequence alignments from a BAM file). Selecting this option from the track settings will replace the visualization of dense data tracks with bar charts that show data coverage. This graph can be seen in tracks such as gene prediction tracks (e.g., RefSeq Genes and UCSC Genes), alignment tracks (e.g., GenBank mRNAs), and cross-species chained alignment tracks. The density coverage graph can also be configured for visibility in custom tracks, track hubs and assembly hubs with supported formats.

#### New keyboard shortcuts

From the Genome Browser data tracks webpage, pressing the question mark ‘?’ key will open a menu of keyboard shortcuts, which has been expanded to include several new options (e.g., toggling zoom views, navigating upstream or downstream in a chromosome, hiding all tracks and launching the ‘view DNA sequence in external tools’ feature).

#### New browser menu options

There have been three major changes to the browser's site-wide top menu bar. First, the ‘Genomes’ menu option now allows quick access to the latest two human and mouse assemblies. Secondly, the ‘View > In External Tools’ menu provides an option to export the current browser region to external tools (e.g., Ensembl, NCBI MapView (37), CRISPOR (38)). Lastly, the ‘Genome Browser’ menu now features a ‘Configure’ link to set visibilities for all tracks on the currently browsed assembly. The ‘Reset All User Settings’ option in the same menu will immediately restore the UCSC Genome Browser on the current Internet browser to a default state by clearing connections to external hubs, removing loaded custom tracks, and reverting to site-wide default configurations (such as default track views for the hg38 assembly).

#### New features in gene description page

In the Genome Browser tracks display, clicking on a gene from a browser's default gene set will display a gene description page containing links to other tools and databases. From this description page, a chart showing RNA-Seq Expression Data from GTEx (53 Tissues, 570 Donors) is now displayed on the two latest human assemblies. Other changes for the gene description page include updated data and links to the MalaCards human disease database (39) and updated Wikipedia links.

### New sessions gallery and public sessions

One widely used feature of the browser is the ‘Sessions’ tool, which allows the user to save the configuration settings of the current browser view for later viewing and sharing (40). Sessions are now arranged in a table with filtering and ordering options based on session name, primary assembly, and creation date. From the session list, clicking on the session name displays that session in the browser. Session expirations have been eliminated to assist with long-term data sharing.

A new Session Gallery has been added, featuring sample sessions created by the UCSC Genome Browser team, which highlight topics of interest to the genomics community. To date, sessions in the gallery include the display of coding and wobble bases, alt-splicing, evolution, variation and disease, topics derived from commonly asked questions on the browser user support mailing list, and sessions used in browser training workshops.

A new ‘Public Sessions’ page has been released, displaying Genome Browser sessions that users have opted to share to this publicly accessed list. In the Session tool's listing of saved sessions for a user's account, a checkbox can be marked to enable public viewing permissions, thus adding the session to the public list. From the Public Sessions page, sessions can be filtered by assembly, name, popularity, creation date, or a phrase from the description. Public Sessions can be reached from the site-wide menu (My Data > Public Sessions).

### Table-based browsing and querying

There are several major interactive web-based tools purposed for browsing and querying underlying tables in the UCSC Genome Browser, including most track data.

First, and most widely used, the web-based Table Browser tool (41) can be accessed directly from the Genome Browser menu, pre-loading current browser configurations for the querying, intersecting, filtering and downloading of genomic data. Another tool is the web-based Data Integrator (42), specializing in combining and exporting data from multiple tracks simultaneously. The Variant Annotation Integrator (VAI) (42) annotates user-provided variant calls with predicted functional effects and data from other tracks.

#### Related database tables in the data integrator

Several UCSC-hosted tracks use multiple related tables in the SQL database to store track data. For many years, the Table Browser has allowed related tables to be joined with the main track table so that their fields can be included in the output. For example, protein product accessions and descriptions associated with RefSeq Genes can be joined by selecting the protAcc and product fields from the refLink table. The Data Integrator now offers the same functionality via the ‘Choose fields…’ button and dialog box.

#### Updated dbNSFP data in hgVai

For hg38, the VAI now offers selected annotations from version 3.1a of the Database of Non-Synonymous Functional Predictions (dbNSFP), which provides pre-computed scores and predictions of functional significance from a variety of tools.

### Custom tracks, track hubs, assembly hubs and public hubs

The UCSC Genome Browser offers three primary options for viewing custom genomic data in the form of custom tracks, track hubs and assembly hubs. These tools provide methods for publicly sharing or privately viewing custom genome assemblies, large-scale genomic annotations, and data tracks.

The collection of data types supported by the custom track, track hub, and assembly hub tools continues to expand. New supported data types include bigPsl, bigChain and bigMaf (which store alignments, pairwise alignments, and multiple alignments, respectively), and CRAM. CRAM is similar to BAM, but denser, using an external reference sequence index. Another new UCSC-specific file type, longTabix, is now supported as a custom track or track hub. This format stores chromatin interactions of paired chromosomal regions. Regional pairs are visualized within the browser with an ‘arc’ representation, similar to chromatin interactions displayed in the WashU EpiGenome Browser (43). For a complete listing of data types supported by track hubs and custom tracks, see Supplementary Table S2.

#### Custom tracks

The simplest way to view custom data against a UCSC-hosted genome assembly is by uploading a file to the browser as a custom track in one of many supported file formats, some of which are new as of this last year (Supplementary Table S2). For most big* compressed formats (e.g., bigWig), the URL to the hosted files alone can be pasted into the custom tracks data-loading dialog box, without additional track information needed.

As with other major tools such as the Table Browser, custom tracks are now integrated with the Data Integrator tool. The new option to view dense data as a bar graph is available for supported custom track data types.

#### Track hubs

Track hubs (44) are web-accessible directories of genomic data that can be viewed as a data portal through the UCSC Browser. The new option to view dense data as a bar graph is available for supported data types.

#### Public UCSC-reviewed track and assembly hubs

The UCSC Genome Browser team continues to promote the use of public track and assembly hubs to display large data sets from consortia and external labs. As of September 2016, there are over 45 public hubs linked for display in the UCSC Genome Browser. Of these, thirteen were added this last year. For a complete listing of recently added public hubs, see Table 2.

**Table 2.** New track and assembly public hubs hosted by UCSC, 2015–2016

Hub name & assemblies	Source
GTEx RNA-Seq Signal Hub: RNA-seq read coverage in 53 tissues from GTEx V6 (7572 samples). *Assemblies: hg38, hg19*	GTEx Consortium, UCSC Computational Genomics Lab, UCSC Genome Browser group
GTEx Analysis Hub: Allele-Specific Expression in 53 Tissues. *Assemblies: hg38, hg19*	GTEx Analysis Working Group (Lappalainen lab, NY Genome Center)
Peptide evidences CNIO: Peptides detected from a re-analysis of multiple experiments and databases. *Assemblies: hg38, hg19*	Spanish National Cancer Research Centre (CNIO), Spanish Institute of Bioinformatics
Principal Splice Isoforms APPRIS: Selects a single CDS variant for each gene as the ‘PRINCIPAL’ isoform based on the range of protein features. *Assemblies: hg38, hg19, mm10, danRer10, rn6, susScr3, panTro4, dm6, ce10*	Spanish National Cancer Research Centre (CNIO), Spanish Institute of Bioinformatics
DASHR small ncRNA: DASHR Human non-coding RNA annotation. *Assemblies: hg19*	Li-San Wang Lab, University of Pennsylvania
Cancer Genomics Tracks: TCGA and ICGC Cancer Mutations, TCGA Expression, Immune Epitopes Database (IEDB), Cancer Immunity Peptides Database, Dienstmann Variant/Cancer database, CIVIC and MyCancerGenome.org. *Assemblies: hg19*	UCSC Genome Browser group
lncRNA in Breast Cancer: long noncoding RNAs in Breast Cancer. *Assemblies: hg19*	Kraus Lab at UT Southwestern
mm9.SMC1.ChIAPET: Cohesin(Smc1)-associated chromatin interactions in murine embryonic stem cells. *Assemblies: mm9*	Young Lab, Whitehead Institute, MIT
Promoterome CAGE and nucleosome positioning: Zebrafish promoterome based on Haberle *et al*. and Nepal *et al*.*Assemblies: danRer7*	Computational Regulatory Genomics Group, MRC Clinical Sciences Centre
Peterhof Yeasts: Assemblies, SNV and CNV data for Saccharomyces cerevisiae strains of the Peterhof Genetic Collection. *Assemblies: sacCer3 & strains*	Saint Petersburg State University
CESAR Gene Mappings to 99 vertebrates: Human Exons mapped by CESAR. *Assemblies: hg38, hg19 > many*	Max Planck Institute of Molecular Cell Biology and Genetics
ChIP-seq data track HUBs from MSC cells from GSE79815. *Assemblies: mm9*	NCBI: Gene Expression Omnibus
UniProt Features: UniProtKB reviewed protein features mapped to the Ensembl genome assembly. *Assemblies: hg38*	The UniProt Consortium

### Alternative UCSC genome browsers: GBiB, mirrors

The UCSC Genome Browser is available as a stand-alone virtual machine, the Genome Browser in a Box (GBiB) (45), that can be used on a personal computer. GBiB provides a complete solution for browsing confidential data, while requiring a small memory footprint. Alternatively, the Genome Browser can be fully or partially mirrored at a third-party site, thus allowing customizations of any browser component or data. The ‘My Data’ menu now includes a new GBiB Shared Data Folder, providing navigation to locally shared files that can be entered as URLs in the custom tracks or track hubs tools. The GBiB custom track tool provides local file-loading capabilities by pointing to the ‘/folders’ directory with the bigDataUrl parameter.

A new supported Genome Browser mirror (*genome-asia.ucsc.edu*) has been released to provide an access point of faster functionality for those users who are geographically closer to Asia than to the United States. This UCSC-administered mirror site is physically located at the RIKEN Yokohama Campus in Japan. Technical support continues to be provided for full and partial mirrors of the Genome Browser offered by academic and noncommercial institutions. Mirror sites can now specify the location of a Galaxy (46) instance to the Table Browser.

### Public MySQL server access

As an alternative to the graphical Table Browser tool, the UCSC-hosted MySQL database is publicly accessible and synchronized on a weekly basis from the main databases on the UCSC Genome Browser website. Many of the command-line utilities from the source tree can be used with the public MySQL databases. These data are stored on a central file server and MySQL server, totaling approximately 20 terabytes of disk space, and growing daily.

This past year, additional security was implemented in the form of SSL (Secure Sockets Layer) connectivity between the browser hosts and MySQL servers. Also, the location of related tables that support the GenBank and RefSeq tracks (e.g., RefSeq, Other RefSeq, mRNA, EST) have moved from individual assembly databases to one shared metadata database, hgFixed.

### Utilities and source code

The UCSC Genome Browser team provides ∼250 application binaries built for standalone command-line use on Linux and Mac OSX platforms. There are several new additions to the command-line utility collection, including file conversion tools (pslToBigPsl, bigPslToPsl, chainToPslBasic) and utilities for loading specific file types into a database (hgLoadChain, hgLoadMaf, hgLoadNet, hgLoadMafSummary).

The entire source code tree, which is written primarily in C, HTML, and Javascript, is publicly available. To install the package, consult the detailed README files in the source tree (http://genome-source.cse.ucsc.edu/gitweb/).

Both the binaries and source code are available for download. Licenses are required for commercial use (https://genome-store.ucsc.edu/), with several exceptions (see http://genome.ucsc.edu/license/).

### Support, training and documentation

This last year, in-person trainings, workshops and presentations were conducted in the United States and international locations, including Germany, Brazil, South Korea, Japan, Sweden, Spain and Denmark. More information regarding workshop requests can be found online (http://bit.ly/ucscTraining).

Multi-media learning materials continue to be developed, such as user guides (e.g., the new multi-region display), online training videos (e.g., how to view exons only and export exon coordinates), announcements of major new data releases, and new blog posts.

## FUTURE PLANS

The UCSC Genome Browser team will continue to release new genome assemblies for species not yet hosted at UCSC, updates for existing genomes, new and updated annotations, new software and overall site improvements. In the coming year, the new Genome Browser in the Cloud (GBiC) will be released. The GBiC is a cloud-based solution that is similar to the stand-alone virtual machine, GBiB.

The Genome Browser team has been working to provide an NCBI RefSeq Gene composite track that will provide alignments and annotations directly from NCBI in addition to providing UCSC's BLAT (47) alignments of RefSeq sequences and resulting transcript models.

Other planned software changes include new features for the multi-region display, improved track sorting, improved track hub searching, extensions to the GTEx track display, a publications track for the human assembly (hg38), and a CRISPR/cas9 track.

## CONTACT US

All UCSC Genome Browser contact information can be found on the website at http://genome.ucsc.edu/contacts.html.
